# A 10-micrometer-thick hydrogel membranes for robust and conformal bioelectronic interfaces

**DOI:** 10.1093/nsr/nwag254

**Published:** 2026-04-30

**Authors:** Hao Tian, Fangchao Xue, Hanlin Cao, Malcolm Xing, Wen Zeng

**Affiliations:** Department of Cell Biology, Third Military Medical University, China; Department of Cell Biology, Third Military Medical University, China; State Key Laboratory of Trauma and Chemical Poisoning, China; Department of Field Surgery Research, Daping Hospital, Third Military Medical University, China; Department of Cell Biology, Third Military Medical University, China; Department of Mechanical Engineering, University of Manitoba, Canada; Department of Cell Biology, Third Military Medical University, China; State Key Laboratory of Trauma and Chemical Poisoning, China; Jinfeng Laboratory, China

**Figure 1.**
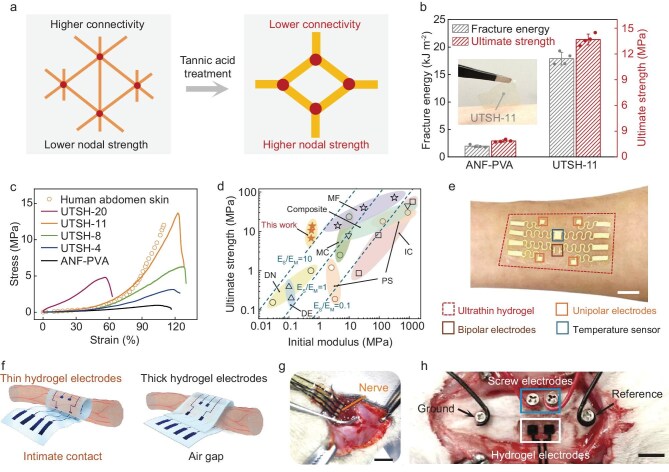
Design, toughening mechanism, and bioelectronic applications of ultrathin ultrastrong hydrogel membranes. (a) Schematic illustration of the structural transition from ANF-PVA to ANF-PVA-TA, showing stronger fibrillar joints and reduced nodal connectivity after incorporation of tannic acid. (b) Fracture energy and tensile strength of ANF-PVA and UTSH-11 hydrogels; inset shows UTSH-11 attached to human skin. (c) Comparison of strain–stress curves for UTSHs with various TA contents (ranging from 0 wt.% to 20 wt.%) and human skin. (d) Strength–softness comparison between UTSHs and representative tough hydrogels. (e) A photograph of wafer-fabricated stretchable electronics integrated on UTSH-11 as an epidermal sensing platform. Schematics (f) and corresponding photographs (g) of UTSH-polypyrrole (PPy) and thick hydrogel-PPy electrodes interfaced with rat sciatic nerves. (h) Optical images of nondestructive UTSH-PPy electrodes and intrusive screw electrodes placed on the cerebral cortex. Reproduced from ref. [2] with permission.

The central challenge in bioelectronics is no longer simply to make devices soft, but to make them mechanically inconspicuous to living tissue while retaining the robustness required for fabrication, implantation, and long-term operation [1]. Materials compliant enough to follow wet, curved, and moving organ surfaces often become fragile when thinned for intimate contact, whereas those reinforced for durability typically lose the compliance needed to avoid interfacial stress. In a recent study in *National Science Review*, Sun *et al.* address this trade-off by developing ultrathin and ultrastrong hydrogel bioelectronic membranes based on self-organized microfibrillar networks (Fig. 1a) [2].

The core innovation lies in structural design. Instead of regarding softness and toughness as contradictory properties, the authors regulate the topology of fibrillar hydrogel networks to achieve biomimetic tissue mechanics. The resulting material exhibits low initial stiffness, obvious strain stiffening, high strength, and excellent fracture toughness. Such nonlinearity is central to biological mechanics. Soft tissues remain compliant under small physiological deformation, yet resist damage at larger strain. By reproducing this behavior in hydrogel membranes only ∼10 μm thick, the study moves closer to the mechanical requirements of truly organ-conformal electronic substrates (Fig. 1b and c).

Equally important is the proposed mechanism. Simulations reveal that the exceptional mechanical performance arises from topological reconfiguration at fibrillar joints within the three-dimensional network, allowing stress to be redistributed under load rather than concentrated locally. This represents more than an incremental material’s improvement. In many reinforced soft materials, strength is achieved through dense fillers or high crosslinking, usually at the cost of flexibility and interfacial comfort (Fig. 1d). Here, toughness instead emerges from how the network reorganizes stress under deformation. For implantable systems, this distinction is crucial: long-term failure is often driven not only by rupture, but also by persistent mechanical mismatch at the tissue interface [3].

The platform is also technologically versatile. The ultrathin membranes can integrate conducting polymers and wafer-fabricated stretchable sensors, enabling multifunctional bioelectronic membranes for multimodal sensing and stimulation. These devices form robust, functional, and biocompatible interfaces with three-dimensional soft organs and tissues, suggesting opportunities in epidermal electronics, brain–machine interfaces, and peripheral nerve interfaces (Fig. 1e–h) [2]. As bioelectronic systems become more sophisticated, the limiting factor is often not the electronics themselves, but the ability to maintain stable, low-stress coupling with complex living tissues over time [4]. A membrane that survives handling and implantation, yet is thin and compliant enough to effectively ‘disappear’ onto tissue, could therefore reshape the design logic of chronic biointerfaces.

More broadly, this study reflects a maturing direction in soft bioelectronics: away from the simple pursuit of lower modulus or greater stretchability, and toward materials whose mechanics are biologically intelligent. Biogels have long attracted attention because they resemble living matter in composition and transport properties [5]. The authors have also developed environmentally stable ionogels with excellent mechanical properties, enabled by the regulation of nanoscale interfacial interactions, further expanding the design space of soft bioelectronic materials [6]. These works show that, with the right architecture, they can also begin to resemble living tissue in how they bear load. That may prove to be the more consequential advance.

Inspired by this work, further development of ultrathin hydrogel bioelectronic interfaces could focus on scalable fabrication, long-term physiological stability, and improved integration with biodegradable or self-healing components. Future research may also explore advanced manufacturing strategies to better adapt these membranes to complex, dynamic tissues for reliable chronic implantation. These efforts will promote the clinical translation of high-performance soft bioelectronic systems.
